# Oscillatory Correlates of Selective Restudy

**DOI:** 10.3389/fnhum.2021.679823

**Published:** 2021-06-11

**Authors:** Michael Wirth, Bernhard Pastötter, Karl-Heinz T. Bäuml

**Affiliations:** ^1^Department of Psychology, Regensburg University, Regensburg, Germany; ^2^Department of Psychology, University of Trier, Trier, Germany

**Keywords:** restudy, context, context retrieval, EEG, brain oscillations

## Abstract

Prior behavioral work has shown that selective restudy of some studied items leaves recall of the other studied items unaffected when lag between study and restudy is short, but improves recall of the other items when lag is prolonged. The beneficial effect has been attributed to context retrieval, assuming that selective restudy reactivates the context at study and thus provides a retrieval cue for the other items (Bäuml, 2019). Here the results of two experiments are reported, in each of which subjects studied a list of items and then, after a short 2-min or a prolonged 10-min lag, restudied some of the list items. Participants' electroencephalography (EEG) was recorded during both the study and restudy phases. In Experiment 2, but not in Experiment 1, subjects engaged in a mental context reinstatement task immediately before the restudy phase started, trying to mentally reinstate the study context. Results of Experiment 1 revealed a theta/alpha power increase from study to restudy after short lag and an alpha/beta power decrease after long lag. Engagement in the mental context reinstatement task in Experiment 2 eliminated the decrease in alpha/beta power. The results are consistent with the view that the observed alpha/beta decrease reflects context retrieval, which became obsolete when there was preceding mental context reinstatement.

## 1. Introduction

Selective retrieval practice can cause forgetting. When subjects study a list of items and, after a short lag, are asked to selectively retrieve a subset of these items, then recall of the remaining unpracticed items is typically impaired relative to a no-practice control condition (e.g., Anderson et al., 1994; Anderson and Spellman, 1995; Bäuml and Kliegl, 2017, for a review). This detrimental effect of selective retrieval, termed retrieval-induced forgetting, has often been attributed to inhibitory processes, assuming that during practice the not-to-be-practiced items interfere and are reduced in strength to attenuate the interference (e.g., Anderson, 2003). The detrimental effect of selective retrieval is retrieval specific, meaning that selective retrieval, but not selective restudy of the same items, induces forgetting of the remaining list items (Anderson et al., 2000; Rupprecht and Bäuml, 2016).

However, selective retrieval practice can also improve recall of other studied items. When subjects study a list of items and after a prolonged lag of 10 min, 30 min, or even days, selectively retrieve some of the studied items, then recall of the remaining items is often improved relative to a no-practice control condition (Bäuml and Dobler, 2015; Wallner and Bäuml, 2017). In contrast to the detrimental effect of selective retrieval, this beneficial effect is not retrieval specific and also arises when there is selective restudy of some studied items (Bäuml and Dobler, 2015; Wallner and Bäuml, 2017). The beneficial effect has been attributed to context retrieval, assuming that, after prolonged lag between study and selective item repetition, the repetition of some of the studied items—be it via retrieval or restudy—reactivates the study context, thus providing a retrieval cue for the remaining items and enhancing their recall performance (see Bäuml, 2019).

The pattern of detrimental and beneficial effects of selective retrieval has been explained by a two-factor framework, in which it is assumed that, in general, selective retrieval induces both inhibition and context retrieval (see Bäuml, 2019). According to this framework, the relative contributions of the two processes depend on the contextual overlap between study and selective retrieval. If the contextual overlap is high - as may occur after a short lag between study and retrieval, during which temporal context will not show much change–then interference between items may be high and inhibition may operate to reduce the interference. Because there will not be much need for context retrieval, as a net result, selective retrieval will impair recall of the unpracticed items. In contrast, if the contextual overlap is low—as may occur after a longer lag between study and retrieval, during which temporal context may drift (Estes, 1955; Mensink and Raaijmakers, 1988)—interitem interference may be reduced and so be the role of inhibition, whereas selective retrieval will trigger context retrieval. As a net result, recall of the remaining items will be enhanced. Because, in general, restudy does not induce inhibition, selective restudy should trigger context retrieval only and do so mainly if the contextual overlap is low.

Both functional magnetic resonance imaging (fMRI) and electroencephalography (EEG) studies have investigated the neural correlates of the detrimental effect. These studies revealed a critical role of prefrontal cortex for this effect (Johansson et al., 2007; Kuhl et al., 2007), showing that this area can suppress cortical patterns unique to the interfering unpracticed items (Wimber et al., 2015). EEG studies examining oscillatory correlates of the forgetting revealed a role of theta oscillations (5–9 Hz) for the effect, the source of which was located in the anterior cingulate cortex and likely reflected modulation of interference during retrieval practice (Staudigl et al., 2010). In another study, practiced and unpracticed items were stored in separate brain hemispheres and selective retrieval of the practiced items was accompanied by increased alpha/beta power (11.5–20 Hz) over the hemisphere housing the sensory representation of the unpracticed items (Waldhauser et al., 2012).

To date, neural correlates of the beneficial effects of selective retrieval have been studied in a single fMRI study only (Jonker et al., 2018). In this study, subjects studied scene-object pairs, with two objects each sharing a common scene stimulus. After a lag, subjects selectively retrieved or restudied one of the two objects associated with each scene. Subjects recalled all previously studied objects on a later test, given the scene contexts as retrieval cues. Both selective retrieval and selective restudy led to increased activations in the posterior medial network as well as in the posterior hippocampal regions. Reactivation of the contextually linked object, however, was constrained to the posterior medial network, and it was most pronounced in a parietal subnetwork, likely reflecting the operation of context retrieval. There are no EEG studies yet examining the beneficial effects of selective retrieval or restudy.

There is one EEG study, however, in which oscillatory correlates of the spacing effect were examined, which has been attributed to context retrieval as well. The spacing effect refers to the observation that repetition of an item during study leads to better recall of the repeated item when repetition is done spaced—with other list items intervening between an item's first and second presentation—rather than massed—when the first and second presentations occur in the absence of any intervening items (Greene, 1989; Kahana, 1996). In this study, van Strien et al. (2007) found that, compared to new words, massed repetitions of study items were accompanied by an increase in theta power (4–6 Hz), whereas spaced repetitions were accompanied by a decrease in alpha power (10–12 Hz). These researchers suggested that the observed increase in theta power reflects an increase in strength of the repeated items (Klimesch et al., 2006), whereas the observed decrease in alpha power reflects “higher processing demands” instead of item-specific information processing. Because the effect of spaced repetition has been attributed to context retrieval – assuming that during spaced repetition the distinct context of the first presentation is retrieved and integrated into the current context representation (Lohnas et al., 2011; Siegel and Kahana, 2014)^1^ – the observed decrease in alpha power may reflect context retrieval.

This study reports the results of two EEG experiments designed to identify oscillatory correlates of selective restudy after short vs. long lag between study and restudy. In each experiment, subjects learned a list of 30 unrelated items, and then restudied 20 of these items, after either a short 2-min or a longer 10-min lag. Finally, a recall test was conducted in which subjects were asked to recall all previously studied items. Participants' EEGs were recorded during the study and restudy phases of each experiment. Oscillatory power was analyzed over different frequency bands and compared between the two lag conditions. Because selective restudy typically has no influence on recall of the other items after short lag but improves recall after longer lag (Bäuml and Dobler, 2015; Wallner and Bäuml, 2017), we expected on the basis of the two-factor framework that differences in oscillatory correlates between lag conditions would point to neural markers of context retrieval. In Experiment 2, but not in Experiment 1, subjects engaged in a mental context reinstatement task immediately before the restudy phase started. Engagement in such a task can lead to mental reinstatement of subjects' study context (Sahakyan and Kelley, 2002; Jonker et al., 2013) and thus may make restudy-induced context retrieval obsolete. A comparison of results between experiments may thus bolster interpretation of results in Experiment 1.

## 2. Experiment 1

### 2.1. Method

#### 2.1.1. Participants

Twenty-four students (15 female, 9 male) from the Regensburg University and the OTH (technical college) of Regensburg took part on a voluntary basis in Experiment 1. Ten additional participants were excluded from the analysis because their EEG data showed too many artifacts or bad electrodes (see exclusion criteria). These participants were replaced by 10 new participants. Sample size followed prior EEG studies that employed permutation-based cluster analysis to examine oscillatory power changes in the areas of selective retrieval and the spacing effect (e.g., van Strien et al., 2007; Hanslmayr et al., 2010; Staudigl et al., 2010) as well as prior behavioral work that studied effects of selective retrieval (e.g., Bäuml and Schlichting, 2014). Participants' mean age was 23.7 years (*SD* = 3.7), ranging from 18 to 34 years. All participants reported normal or corrected-to-normal vision. All participants spoke German as their native language. No participant reported any history of a diagnosed psychological or neurological disease. All participants gave written informed consent and were reimbursed 13 Euros after participation.

#### 2.1.2. Materials

The stimuli were 60 unrelated, concrete German nouns drawn from the CELEX database using the WordGen software toolbox (Version 1.0, Duyck et al., 2004). Two lists of 30 words each, containing 10 target items and 20 nontarget items were constructed and balanced for their taxonomic frequency and word length; all target items of a list began with a unique first letter and all nontarget items of a list had a unique two-letter word stem. Picture sets of 50 foods and 50 vacation destinations were taken from Wallner and Bäuml (2017) and used for rating tasks before the study phases of the short-lag and long-lag blocks.

#### 2.1.3. Design

**Figure 1:**
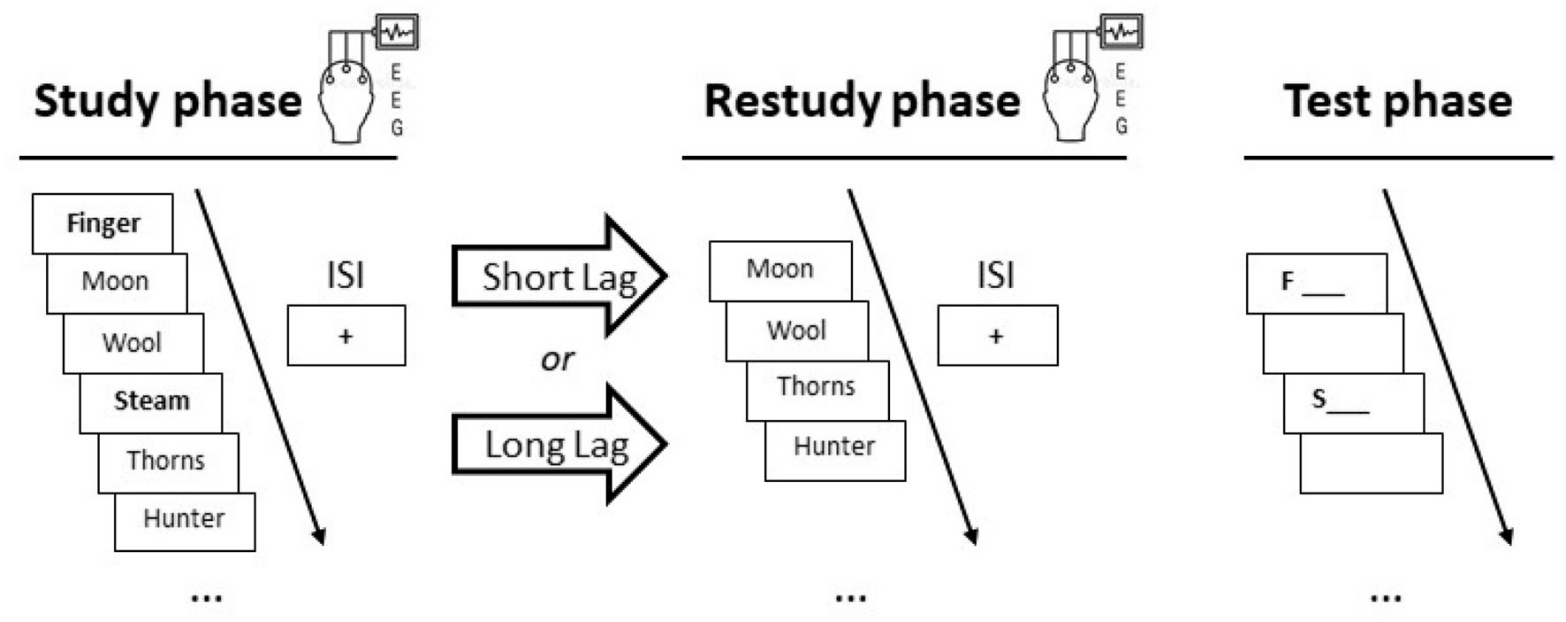
Procedure in the two experiments. In each experiment, participants took part in two experimental blocks: one block with a short 2-min lag between study and restudy, and another block with a prolonged 10-min lag between the two phases. In both blocks, participants studied a list of unrelated items, restudied some of the list items after a (short or long) lag, and recalled the remaining (target) items (printed in bold letters) in the final test phase. Scalp EEG was recorded during the study and restudy phases. In Experiment 2, participants engaged in a mental context reinstatement task immediately before the restudy phase started, to mentally reinstate the study context (see section 3.1.3 for details). ISI = inter-stimulus interval.

The only variable that was manipulated was lag (short, long) between study and restudy phases (see Figure 1). Regarding behavior, the target recall rate was examined as a function of lag (short vs. long). Regarding electrophysiology, stimulus-induced EEG power changes during the encoding of items were examined as a function of phase (study, restudy) and lag (short vs. long). The rating task was included to increase contextual dissimilarity between experimental blocks and make the procedure comparable to Experiment 2.

#### 2.1.4. Procedure

After preparation for EEG data collection (see below), participants completed two experimental blocks, i.e., a short-lag block and a long-lag block, with the order of the two blocks counterbalanced between participants. Each block started with a picture-rating task. Either 50 pictures of foods or 50 pictures of vacation destinations were shown. Half of the participants rated the food pictures in the short-lag condition, the other half in the long-lag condition. Each picture was presented for 6 s each and participants were asked to rate on a 6-point rating scale how much they would like to eat this food or how much they would like to spend their vacation at this vacation destination (1 = very much, 6 = not at all).

Scalp EEG was recorded during the study and restudy phases. At the beginning of each block, participants were instructed to study items for an upcoming recall test. In the study phase, the 30 items of a list (10 target items, 20 nontarget items) were presented successively and in random order with an item-presentation time of 5 s. Items were shown in the center of the screen. Between item presentations in the inter-stimulus interval (ISI), a fixation cross was shown in the center of the screen. The ISI had a variable duration of 1.3–1.7 s. Experimental blocks differed in what happened after the study phase. In the short-lag block participants counted backwards for 120 s, whereas in the long-lag block, participants alternated between solving arithmetical problems and engaging in two mental context change tasks for 600 s (see Wallner and Bäuml, 2017). During the context change tasks, participants were asked to close their eyes to imagine a scenario for 45 s, followed by a 120 s window to write down their imaginations. Two out of four possible context change tasks were randomly drawn for each participant. The four possible tasks were “imagination of parents' house” (see Sahakyan and Kelley, 2002), “imagination of an international vacation” (see Delaney et al., 2010), “imagination of living in the Middle Ages”, and “imagination of way to first school”. The combination of arithmetical problems and mental context change tasks has been used to produce behavioral context effects that are similar to those typically observed after a delay of 1 day (see Wallner and Bäuml, 2017).

The restudy phase consisted of two restudy cycles. At the beginning of each cycle, participants were told that they would now get the opportunity to restudy a subset of the originally studied items for the upcoming recall test. In each cycle, a list's 20 nontarget items were presented in the center of the screen in random order with item-presentation time of 5 s. The presentation time of the fixation cross in the ISI was again variable between 1.3 and 1.7 s. Immediately after the second restudy cycle, participants recall for the target and nontarget items was assessed in the test phase. Participants were asked to verbally recall the 10 target items first and the 20 nontarget items second. Target items were cued with their unique first letter, whereas nontarget items were cued with the first two letters. All cues were presented in the center of the screen with a presentation time of 6 s. The ISI was constant with 0.5 s.

#### 2.1.5. Recording of EEG Data

Scalp EEG was recorded from 61 equidistant active electrodes mounted in an elastic cap (Acticap, Montage 10, Brain Products, Gilching, Germany). Electrode Cz served as the common reference. Vertical and horizontal eye movements were recorded from two additional face electrodes. Electrode-skin impedance was kept below 20 kΩ. Signals were digitized with a sampling rate of 500 Hz and amplified between 0.3 and 1,000 Hz with a notch filter at 50 Hz to remove power-line noise (BrainAmpMR plus, BrainVision Recorder, v1.21, Brain Products, Gilching, Germany).

#### 2.1.6. Exclusion Criteria

Participants with more than nine interpolated channels, participants with <15 artifact-free study trials (2.5 s interval around stimulus onset), and participants with <15 artifact-free restudy trials in each of the two restudy blocks (2.5 s interval around stimulus onset) were excluded and replaced by new participants (see Hanslmayr et al., 2009, for a simulation experiment showing that EEG oscillatory power changes stabilize with 15 segments per condition). For the final sample that went into analysis, the mean number of interpolated channels was 6.5 (*SD* = 1.9); the mean numbers of trials included in the analysis were 27.5 (*SD* = 2.2) study trials in the short-lag condition, 27.2 (*SD* = 2.6) study trials in the long-lag condition, 36.9 (*SD* = 2.7) restudy trials in the short-lag condition, and 36.6 (*SD* = 2.1) restudy trials in the long-lag condition.

#### 2.1.7. Time-Frequency Decomposition

EEG data were transformed into the time-frequency domain using a complex demodulation algorithm, which is implemented in BESA Research (v5.3 Hoechstetter et al., 2004). The algorithm consists of a multiplication of the time-domain signal with a complex periodic exponential function, having a frequency equal to the frequency under analysis, and subsequent low-pass filtering. The low-pass filter is a finite impulse response filter of Gaussian shape in the time domain, which is related to the envelope of the moving window in wavelet analysis. The data were filtered in a frequency range from 2 to 20 Hz. Time resolution was set to 78.8 ms (full power width at half maximum; FWHM), and frequency resolution to 1.42 Hz (FWHM). Time-frequency data were exported in bins of 50 ms and 1 Hz.

#### 2.1.8. Analyses of EEG Power Changes

Stimulus-induced power changes were determined by calculating temporal-spectral evolution, i.e., power changes during word presentation for all time-frequency points with power increases or decreases at each time point and frequency f related to mean power at frequency f over the prestimulus baseline interval (Pfurtscheller and Aranibar, 1977; Pfurtscheller and Da Silva, 1999). The baseline interval was set from –0.5 s to stimulus onset. Permutation-based cluster analysis (Maris and Oostenveld, 2007) was calculated to examine the interaction between phase (study, restudy) and lag (short, long), as implemented in BESA Statistics (v2.1, BESA Software). If the interaction turned out to be significant, simple main effects of phase (study vs. restudy), separately for short and long lag conditions, were examined. Alternatively, if the interaction turned out to be not significant, the main effect of phase (study vs. restudy) was examined independent of lag.

Two steps were taken in the statistical analysis of the time-frequency data. In the first step, a non-spatial cluster analysis was calculated, in which time-frequency spectrograms of power changes were averaged across the 61 electrodes. Regarding the interaction effect between phase (study, restudy) and lag (short, long), difference scores of averaged power changes (restudy minus study) were contrasted in single *t*-tests between short and long lag conditions. Regarding the main effect and simple main effects of phase (study vs. restudy), averaged power changes were contrasted in single *t*-tests between study and restudy phases independent of (main effect) or separately for (simple main effects) the two lag conditions. Because context reactivation effects were expected within the first second of item presentation time (van Strien et al., 2007), cluster analyses were calculated for the time range from 0 to 1 s after stimulus onset (we note however that additional exploratory cluster analysis of the later time range from 1 to 2 s following stimulus onset revealed no significant empirical cluster for the phase *x* lag interaction). Specifically, in the cluster analyses, two-tailed *t*-tests were calculated for all time-frequency points (21 [50 ms time bins from 0 to 1 s] * 19 [1 Hz frequency bins from 2 to 20 Hz]) and clusters of contiguous significant data points (*p* < 0.05 in the *t*-test) were derived. For each empirical cluster, the sum of *t*-values of the single significant data points was kept as a test statistic. Random permutation tests (5,000 runs) were run in which the sum test statistic was repeatedly calculated for randomly shuffled data sets, with the data randomly reordered across short and long lag conditions, and the permutation-based cluster with the highest sum of *t*-values was kept. Test statistics for empirical clusters were compared to the null distribution of the permutation-based clusters and a p_*clust*_ value for each empirically derived cluster was calculated. Descriptive statistics (mean power changes and standard errors) were calculated for each significant non-spatial cluster by averaging across data points of the cluster's maximum time range and maximum frequency range (see Table 1).

**Table 1:** Mean percent power changes (and standard errors) for each significant non-spatial cluster per condition.

		**Non-spatial cluster**		**Short lag**		**Long lag**	
**Experiment**	**Figure**	**Time**	**Frequency**	**Study**	**Restudy**	**Study**	**Restudy**
Exp. 1	2A	0–750	7–19	–7.7(2.6)	–3.9 (2.6)	–5.5 (2.6)	–10.4 (2.4)
	2B	300–700	7–14	–15.5 (3.9)	–9.1 (4.3)		
	2C	50–350	9–11			–0.6 (4.3)	–7.4 (4.2)
	2C	100–650	14–20			–7.1 (2.3)	–12.6 (2.0)
Exp. 2	3B	100–250	6–8	20.4 (6.0)	24.9 (5.0)	18.5 (6.0)	25.6 (5.8)
	3B	450–550	4–6	14.0 (4.3)	18.5 (4.4)	12.0 (4.5)	17.2 (5.2)
	3B	600–700	16–17	–3.0 (4.9)	–8.5 (3.0)	–3.0 (4.2)	–8.5 (3.1)
	3B	650–800	12–13	–8.3 (5.5)	–14.2 (4.7)	–8.6 (5.5)	–15.6 (3.8)

*Means were obtained by averaging across all data points of the cluster's maximum time range and maximum frequency range. Time in ms, frequency in Hz*.

In the second step, empirical clusters with a *p*_*clust*_ value below 0.05 went into spatial analysis. For clusters showing effects in the same direction, i.e., clusters with positive or negative t sum test statistics, power changes were averaged across data points of the clusters composed time range and composed frequency range (including non-significant time-frequency points between clusters), separately for each electrode. Two-tailed *t*-tests were calculated for all electrodes. Spatial topographies were identified by considering those electrodes that were significant in the *t*-test. No cluster-permutation tests were calculated within spatial analyses. Thus, both clustered and scattered spatial effects were considered. Note that the present two-step analysis was chosen to give less weight to differences in topography between conditions. In fact, cortical networks related to memory (re)activation in general and context reactivation in particular should be distributed over the scalp. Accordingly, a one-step analysis, in which time, frequency, and electrode are considered simultaneously in the permutation test, would consider distributed effects with the same time-frequency characteristics as different clusters. Similar two-step procedures were also used by the authors in earlier work (e.g., Pastötter and Bäuml, 2014, 2016).

### 2.2. Results

#### 2.2.1. Behavioral Results

Participants recalled 46.7% (*SE* = 4.2%) of the target items in the short lag condition and 48.8% (*SE* = 5.1%) in the long lag condition. The difference between conditions was not significant, *t*_(23)_ < 1, which is consistent with prior studies demonstrating that selective restudy after prolonged lag can abolish lag-induced forgetting (Wallner and Bäuml, 2017). Nontarget recall rates were 82.7% (*SE* = 1.5%) in the short lag condition and 81.0% (*SE* = 2.6%) in the long lag condition, *t*_(23)_ < 1.

#### 2.2.2. Electrophysiological Results

**Figure 2:**
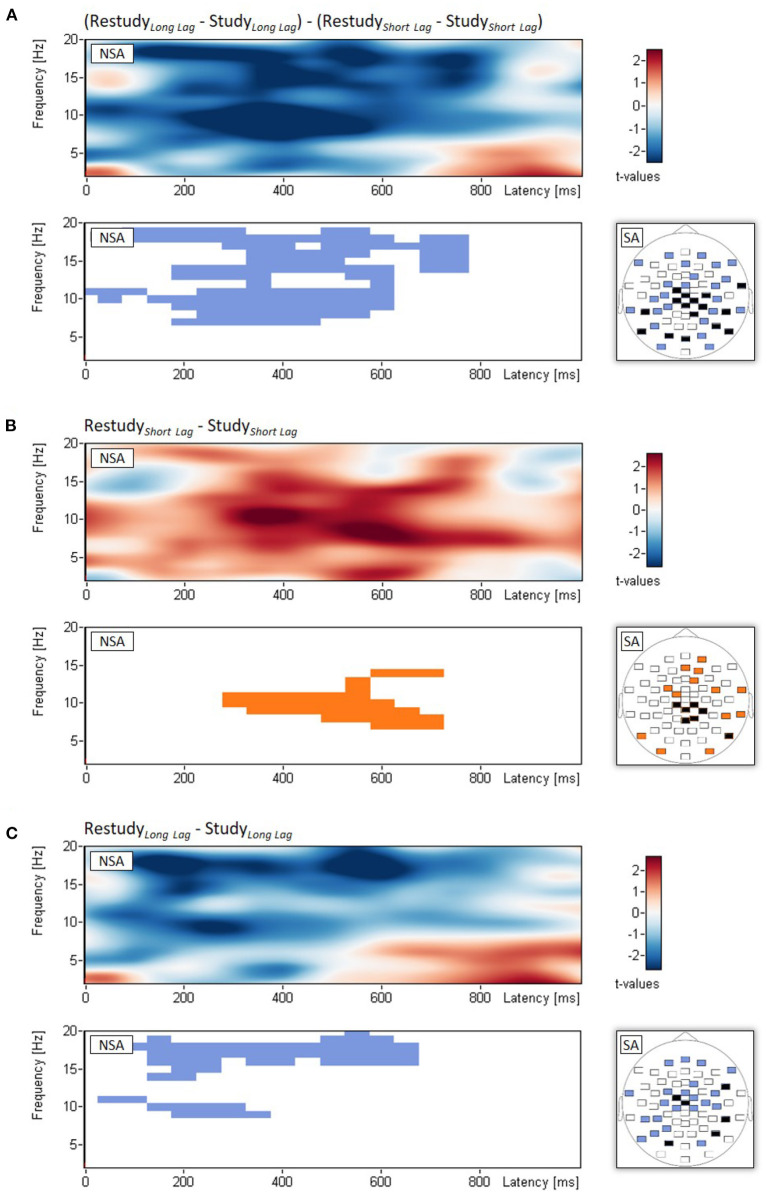
EEG results of Experiment 1. Non-spatial analyses (NSA): Time-frequency spectrograms of *t*-values and significant clusters for **(A)** the two-way interaction (examined with *t*-tests on difference scores) between phase (study, restudy) and lag (short, long), **(B)** the simple main effect of phase for the short-lag condition, and **(C)** the simple main effect of phase for the long-lag condition. **(A–C)** Spatial analyses (SA): Blue and orange electrodes: *p* < 0.05, black electrodes: *p* < 0.01. Note that for each topographical plot, all differences of the significant electrodes were in the same direction.

Non-spatial cluster analysis, which tested the interaction between phase (study, restudy) and lag (short, long) with time-frequency spectrograms of power changes averaged across the 61 scalp electrodes, revealed a significant empirical cluster in the alpha/beta frequency range (7-19 Hz), which extended approximately from 0 to 750 ms after stimulus onset, *p*_*clust*_ < 0.001 (see Figure 2A). The interaction effect was neither significantly related to the behavioral difference in target recall between short and long lag, *r* = –0.19, *p* = 0.370, nor to the difference in nontarget recall, *r* = 0.38, *p* = 0.064. Spatial analysis indicated that the interaction effect was most pronounced over central and parietal electrodes (see Figure 2A).

Follow-up analyses of the simple main effects of phase (study, restudy) were calculated separately for the two lag conditions. Regarding the short lag condition, this analysis showed a cluster of power increase from study to restudy in the theta/alpha frequency range (7–14 Hz) that extended approximately from 300 to 700 ms after stimulus onset, *p*_*clust*_ < 0.001 (see Figure 2B). Regarding the long lag condition, the analysis revealed two clusters of power decrease from study to restudy in the alpha (9–11 Hz) and beta frequency range (14–20 Hz), which extended approximately from 50 to 350 ms, *p*_*clust*_ < 0.001, and 100–650 ms after stimulus onset, *p*_*clust*_ = 0.022, (see Figure 2C). Spatial topographies of these simple main effects are shown in Figures 2B,C.

### 2.3. Discussion

The behavioral results are consistent with prior work showing that, for lags in the order of 10 min between study and restudy, restudy of some studied items can eliminate lag-induced forgetting of the list's other (target) items, thus leading to similar target recall levels after short and long lag (Wallner and Bäuml, 2017). Examining oscillatory activities during study and restudy after both short and long lag between study and restudy, the results show an interaction in the alpha/beta frequency range. This interaction is due to a theta/alpha power increase from study to restudy in the short lag condition and an alpha/beta power decrease from study to restudy in the long lag condition. On the basis of the prior work, indicating that after longer lag restudy induces context retrieval (Bäuml and Dobler, 2015; Wallner and Bäuml, 2017), this finding indicates that the observed alpha/beta decrease may reflect context retrieval.

Concluding from the findings of Experiment 1 that the decrease in alpha/beta power reflects context retrieval may be premature, however. Indeed, while it appears likely that the effect reflects some form of memory reactivation, it is unclear whether this reactivation reflects context retrieval, as is suggested by the two-factor framework (Bäuml, 2019), or reactivation of the restudied items themselves. In the literature, (parietal) alpha power decrease has been suggested to be positively related to memory reactivation in both short-term (Klimesch et al., 2005) and long-term memory tasks (Martín-Buro et al., 2020; Griffiths et al., 2021). For instance, Martín-Buro et al. (2020) examined alpha power decrease during recognition testing and found that left posterior alpha power decrease was positively related to both item recognition and associative recall. Griffiths et al. (2021) examined alpha/beta power decrease with recall testing and argued that alpha/beta power decrease may be unrelated to the reactivation of stimulus-specific information but related to “supportive processes” that accompany the encoding and retrieval of episodic memories.

To examine whether the interaction effect observed in Experiment 1 and the simple main effect of decreased alpha/beta power in the long lag condition reflect context retrieval, in Experiment 2 we repeated Experiment 1 with the only difference that prior to the restudy phase, subjects were engaged in a mental context reinstatement task. In such a task, subjects are asked to think back to the study phase and describe the thoughts, feelings, and emotions they had immediately before the experiment started (e.g., Sahakyan and Kelley, 2002; Jonker et al., 2013). After longer lag, such mental context reinstatement has been found to largely eliminate lag-induced context change and thus make recall after long lag comparable to recall after short lag (e.g., Wallner and Bäuml, 2017; Kliegl et al., 2019). Thus, after longer lag, there should no longer be much need for context retrieval if mental context reinstatement preceded the restudy phase. Therefore, if the decrease in alpha/beta power after long lag reflected context retrieval, the decrease should disappear in Experiment 2.

## 3. Experiment 2

### 3.1. Method

#### 3.1.1. Participants

Another 24 students (17 female, 7 male) from the Regensburg University and the OTH of Regensburg took part on a voluntary basis in Experiment 2. Six additional participants were excluded due to heavy artifacts or bad electrodes in the EEG. Participants' mean age was 21.7 years (*SD* = 3.1), ranging from 18 to 30 years. All participants reported normal or corrected-to-normal vision and spoke German as their native language. No participant reported any history of a diagnosed psychological or neurological disease. All participants gave written informed consent and were reimbursed 13 Euros after participation.

#### 3.1.2. Materials and Design

The materials and design were identical to Experiment 1.

#### 3.1.3. Procedure

The procedure was the same as in Experiment 1 with the following exception: backward counting in the short lag condition and the solving of arithmetic problems directly before the restudy phase in the long lag condition were reduced by 1 min. Instead, directly before the first restudy block, participants were asked to mentally reinstate contextual elements of the rating task that preceded the study phase. Specifically, participants were told to recall and write down in as much detail as possible their thoughts, feelings, and emotions that they had experienced while viewing the pictures in the rating task. This method has been shown to successfully reinstate mental context in participants in earlier work (Wallner and Bäuml, 2017).

#### 3.1.4. EEG Data Analysis

The recording, preprocessing, time-frequency demodulation, and calculated time-frequency analyses were identical to Experiment 1.

### 3.2. Results

#### 3.2.1. Behavioral Results

Target recall levels were comparable to those of Experiment 1. Participants recalled 46.3% (*SE* = 4.7%) of the target items in the short lag condition and 42.1% (*SE* = 4.7%) in the long lag condition. The difference between conditions was not significant, *t*_(23)_ < 1. Nontarget recall rates were 80.8% (*SE* = 2.3%) in the short lag condition and 81.0% (*SE* = 2.1%) in the long lag condition, *t*_(23)_ < 1.

#### 3.2.2. Electrophysiological Results

**Figure 3:**
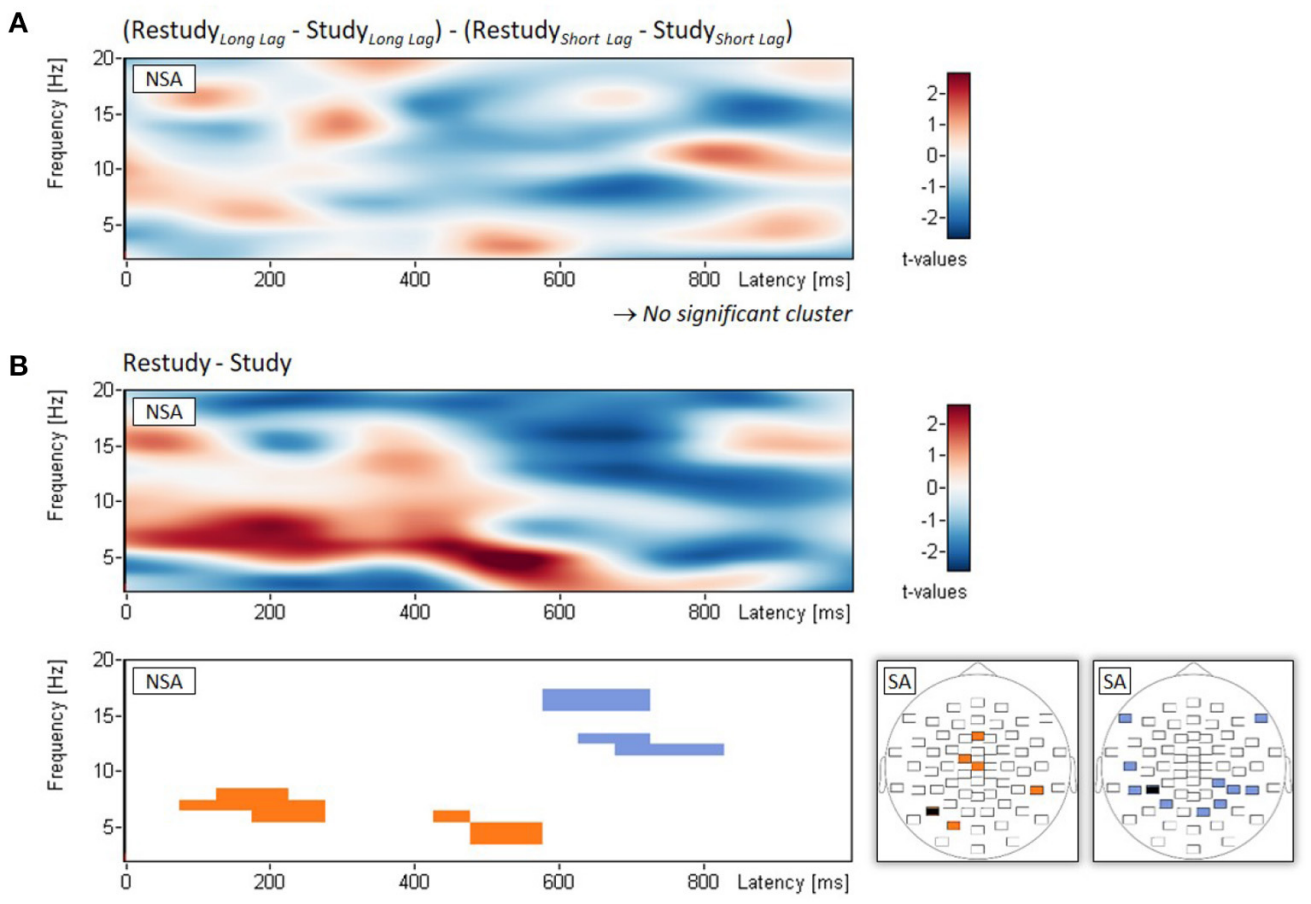
EEG results of Experiment 2. Non-spatial analyses (NSA): Time-frequency spectrograms of *t*-values and significant clusters for **(A)** the two-way interaction (examined with *t*-tests on difference scores) between phase (study, restudy) and lag (short, long), and **(B)** the main effect of phase (study, restudy) independent of lag conditions. Spatial analysis (SA): Blue and orange electrodes: *p* < 0.05, black electrodes: *p* < 0.01. Note that for each of the two topographical plots, all differences of the significant electrodes were in the same direction.

Regarding the interaction between phase (study, restudy) and lag (short, long), non-spatial cluster analysis showed no significant empirical cluster, all *p*_*clust*_ > 0.687 (see Figure 3A). The analysis of the main effect of phase (study, restudy), with power changes averaged across lag conditions, revealed two clusters of power increase and two clusters of power decrease from study to restudy. The two clusters of power increase composed in the theta frequency range (4-8 Hz) and extended approximately from 100 to 550 ms following stimulus onset, *p*_*clust*_ = 0.016 and 0.035, respectively (see Figure 3B). The two clusters of power decrease composed in the alpha/beta frequency range (12-17 Hz) and extended approximately from 600 to 800 ms after stimulus onset, *p*_*clust*_ = 0.034 and 0.049, respectively (Figure 3B). In addition, Figure 3B shows the spatial topographies of these power increases and power decreases from study to restudy phases.

#### 3.2.3. Additional Exploratory Analysis

**Figure 4:**
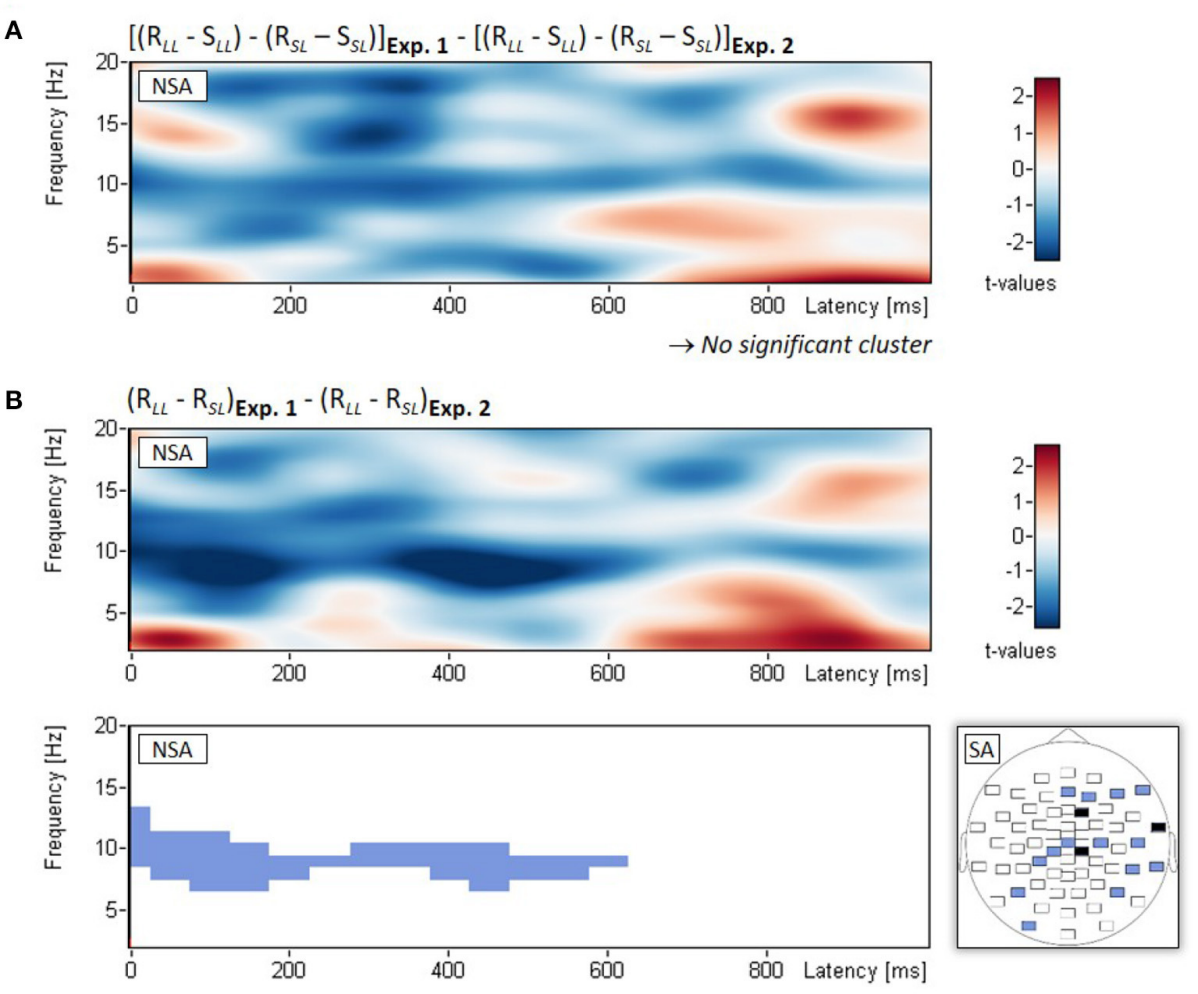
Comparison of Experiments 1 and 2: EEG results from the additional exploratory analysis. Non-spatial analyses (NSA): Time-frequency spectrograms of *t*-values and significant clusters for **(A)** the three-way interaction (examined with *t*-tests on double difference scores) between Experiment (Experiment 1, Experiment 2), phase (study, restudy), and lag (short, long), and **(B)** the two-way interaction (examined with *t*-tests on difference scores) between Experiment (Experiment 1, Experiment 2) and lag (short, long); the latter was calculated for the restudy phase only. Spatial analysis (SA): Blue electrodes: *p* < 0.05, black electrodes: *p* < 0.01; all differences of the significant electrodes were in the same direction.

In two exploratory analyses, we directly compared EEG power changes between the two experiments. First, we examined the three-way interaction between Experiment (Experiment 1, Experiment 2), phase (study, restudy), and lag (short, long). Non-spatial cluster analysis of the three-way interaction (examined with *t*-tests on double difference scores) showed no significant empirical cluster, all *p*_*clust*_ > 0.527 (see Figure 4A). Second, because any differences in lag effects between experiments should arise from the restudy phase, we examined the two-way interaction between Experiment (Experiment 1, Experiment 2) and lag (short, long) with regard to power changes during the restudy of the nontarget items. Non-spatial cluster analysis of the two-way interaction (examined with *t*-tests on difference scores) revealed a significant empirical cluster in the alpha frequency range (7–13 Hz), which extended approximately from 0 to 600 ms after stimulus onset, *p*_*clust*_ = 0.020 (see Figure 4B). The finding indicates a significantly larger decrease of alpha power from short to long lag in Experiment 1 (–9.2%) compared to the smaller difference in alpha power between lags in Experiment 2 (–1.1%). Figure 4B also shows the spatial topography of this two-way interaction effect.

### 3.3. Discussion

In contrast to Experiment 1, examination of the oscillatory activities during study and restudy for the short and long lag conditions showed no significant interaction in the alpha/beta frequency range. Because Experiments 1 and 2 differed only in whether there was mental context reinstatement prior to restudy of the nontarget items (Experiment 2) or not (Experiment 1), these findings indicate that the induced mental context reinstatement eliminated the interaction effect. Prior mental context reinstatement should reduce the need for further restudy-induced context retrieval (Wallner and Bäuml, 2017). The finding therefore suggests that the alpha/beta interaction effect observed in Experiment 1 indeed reflects context retrieval.

The results of Experiment 2 also revealed main effects of phase, indicating differences in activities between study and restudy. From study to restudy, both an increase in theta power and a decrease in alpha/beta power were observed. Whereas, the increase in theta power to some degree resembles the observed increase in theta/alpha power after short lag in Experiment 1, the decrease in alpha/beta power is similar to the observed decrease in roughly the same frequency range after long lag in Experiment 1. Thus, at least in tendency, the effects observed separately for the short and long lag conditions in Experiment 1 appear to be present in Experiment 2 as well, though largely reduced in size due to the induced mental context reinstatement.

## 4. General Discussion

The main result arising from the present experiments is that, after longer lag between study and restudy, restudy of some studied items can induce a decrease in alpha/beta power that, on the behavioral side, is accompanied by the absence of lag-induced forgetting for the not restudied target items. Critically, the observed decrease in alpha/beta power was found to be no longer present when mental reinstatement of the study context preceded the restudy phase. These findings are consistent with the view that the alpha/beta decrease reflects restudy-induced context retrieval that becomes obsolete when there is preceding mental reinstatement of the study context. The alpha/beta decrease may thus represent a neural marker of context retrieval.

The present EEG finding complements the results of the previous fMRI study by Jonker et al. (2018), in which selective restudy has been shown to increase neural activities in the posterior medial network as well as in the posterior hippocampal regions. Importantly, it was the increased activity in the posterior medial network, and here a parietal subnetwork, which reflected the operation of context retrieval. In this prior study, increased activities in the very same areas were also found when selective restudy was replaced by selective retrieval and these activities were even enhanced in response to retrieval vs. restudy. It is a high priority for future work to investigate whether the alpha/beta decrease found in the present study generalizes from restudy to retrieval and is even enlarged when selective item repetition occurs via retrieval.

The present results relate to the previous study by van Strien et al. (2007) on oscillatory correlates of the spacing effect. In this study, van Strien et al. (2007) found that, compared to new words, massed repetitions of study items are accompanied by an increase in theta power (4–6 Hz), whereas spaced repetitions are accompanied by a decrease in alpha power (10–12 Hz). Whereas, the observed increase in theta power was attributed to an increase in strength of the repeated items, the observed decrease in alpha power was attributed to “higher processing demands” instead of item-specific information processing. With the observed increase in theta/alpha power after short lag and the observed decrease in alpha/beta power after longer lag, the present findings show some similarity to this prior work. The observed theta/alpha power increase may thus reflect an increase in memory strength of the restudied items (but see below), whereas the observed alpha/beta power decrease may reflect context retrieval.

In a recent EEG study, Griffiths et al. (2021) showed that, on a trial-by-trial basis, an alpha/beta power decrease during encoding can predict an alpha/beta power decrease during retrieval. The findings suggest that a decrease in retrieval-related alpha/beta power can be contingent on the decrease in alpha/beta power that was found during encoding. Critically, the observed decreases in alpha/beta power were not found to reflect stimulus identity, suggesting that the contingency between encoding-related and retrieval-related alpha/beta power reflects the reactivation of a neurophysiological operation, rather than neural representation. Griffiths et al. (2021) called this neurophysiological operation “supportive processes”. We suggest that the decrease in alpha/beta power during study may reflect the encoding of context and the decrease in alpha/beta power during retrieval the reactivation of study context.

The present finding of an increase in theta/alpha power after short lag between study and restudy parallels findings from prior work on multi-list learning, in which theta power (5–8 Hz) and alpha power (10–13 Hz) were found to continuously increase from a first studied list to the study of a second list and the study of even further lists (Pastötter et al., 2008, 2011; Kliegl et al., 2015). Like in the short lag condition of the present study, lag between study of the single lists was also short in this multi-list learning task. Because increases in theta activity have been linked to memory load (Jensen and Tesche, 2002; Sederberg et al., 2006) and increases in alpha activity to inattention (Palva and Palva, 2007; Klimesch, 2012), these researchers related the observed increase in theta and alpha power to inattentional encoding and increased memory load. Accordingly, the increase in theta/alpha power observed after short lag in the present study may not only reflect an increase in memory strength of the restudied material (see above) but also an increase in inattention or memory load.

The present study does not provide a direct demonstration that, after short lag, restudy leaves recall unaffected, and, after long lag, improves recall performance. Still, the present finding that selective restudy led to similar recall levels after short and long lag is in perfect agreement with the prior literature. In fact, given that restudy has been shown to not influence recall when lag between study and restudy is short (e.g., Anderson et al., 2000; Bäuml and Aslan, 2004) and, in the absence of selective restudy, recall is generally reduced when there is a longer lag of 10–20 min between study and test (e.g., Wallner and Bäuml, 2017; Kliegl et al., 2019), a beneficial effect of restudy after longer lag should lead to similar recall levels after short and long lag (e.g., Wallner and Bäuml, 2017). This similarity in recall level is exactly what we found, thus supporting the view that restudy improved recall after long lag also in the present study. All this holds although the results do not provide a direct demonstration of the effect.

The present study provides first evidence for a specific neural marker of context retrieval in Experiment 1, which was supported by the finding of a lack of this marker in Experiment 2 when there was mental context reinstatement prior to selective restudy. This difference in results between experiments could not be bolstered by a significant three-way interaction of experiment (1, 2) × phase (study, restudy) x lag (short, long), suggesting that too much noise was present in the data due to the employed between-subjects design. Future studies may revisit the issue by increasing the number of participants or by employing a full within-subjects design. Such a within-subjects design could solve possible power issues and may also permit to include a selective retrieval condition, in order to directly compare the effects of retrieval and restudy within a single experiment. Such design may even allow to include an additional control condition, in which selective restudy (and selective retrieval) were replaced by unrelated distractor tasks, which would provide the opportunity to replicate the behavioral beneficial effects of selective restudy (and selective retrieval).

Future research might also want to establish a more direct link between the observed decrease in alpha/beta power and context (retrieval) employing a multivariate pattern analysis (MVPA) approach. Such approach could comprise a study context influenced and characterized by a preceding theme for a distinct temporal context (see Staudigl et al., 2015). Neural pattern classifiers could be built to distinguish the EEG during the two study phases (i.e., context theme 1 vs context theme 2), and could then be generalized and applied to the EEG data recorded in the restudy phases. If the observed decrease in alpha/beta power is indeed linked to context retrieval, one should find a (greater) classification of the study context due to cortical reinstatement in the restudy phase when lag is long and prior mental context reinstatement is absent. However, when employing such MVPA approach in future work, amount of trials should be drastically increased relative to the present study.

*To conclude*, this is the first study to report a putative EEG marker of context retrieval when there is lagged restudy of previously studied items. The finding complements recent fMRI findings on selective retrieval and selective restudy, which indicated a role of parietal regions for context retrieval, and parallels the results of a recent EEG study on the spacing effect, indicating that a decrease in alpha/beta power reflects context retrieval. Following the two-factor framework of the effects of selective item repetition (Bäuml, 2019), the context retrieval interpretation of the observed alpha/beta decrease suggests that the present results may generalize from selective restudy to selective retrieval. Ideally, future work may conduct a combined fMRI-EEG study to (i) replicate the fMRI findings by Jonker et al. (2018) and the EEG findings of the present study, and (ii) additionally show that the results hold for both forms of selective item repetition - repetition via restudy as well as repetition via retrieval.

## Data Availability Statement

The datasets presented in this study can be found in online repositories. The names of the repository/repositories and accession number(s) can be found here: https://osf.io/dr3z5/.

## Ethics Statement

Ethical review and approval was not required for the study on human participants in accordance with the local legislation and institutional requirements. The patients/participants provided their written informed consent to participate in this study.

## Author Contributions

MW, BP, and K-HB developed the study concept and experimental design. All three authors contributed to the writing of the manuscript. MW organized the data collection, MW and BP performed the data analysis. All authors approved the final version of the manuscript for submission.

## Conflict of Interest

The authors declare that the research was conducted in the absence of any commercial or financial relationships that could be construed as a potential conflict of interest.

## Footnotes

^1^After spaced repetition, the integrated context representation of a repeated item should include a richer set of contextual features than after massed repetition, where the context representations of the first and second presentations should be fairly similar and so the integrated context represenation should be less rich in distinct features than after spaced repetition. It is this difference in context representation that is supposed to underlie the difference in recall performance.
